
JOURNAL INFORMATION
==============================
NLM Title Abbreviation: Eur J Gen Pract
Journal ID: Eur J Gen Pract
NLM Journal ID: 9513566

The European Journal of General Practice

ISSN: 1381-4788
EISSN: 1751-1402
Publisher: Taylor & Francis

ARTICLE INFORMATION
==============================
PMCID: PMC10171114
DOI: 10.1080/13814788.2023.2171395
Article ID: 2171395
Article version: 1
Article version: Version of Record
Subjects: Abstract, Abstracts

Research on integrated community care: Focus on general practitioners, patients and the population. Selected abstracts from the 95th EGPRN conference Antwerp, Belgium, 20–23 October 2022

All abstracts of the conference can be found at the EGPRN website https://www.egprn.org/page/conference-abstracts

Abstracts

European Journal of General Practice

Electronic publication date: 2023 May 9
Publication date: 2023
Volume: 29
Issue: 1
Electronic Location ID: 2171395
Copyright: © 2023 The Author(s). Published by Informa UK Limited, trading as Taylor & Francis Group.
Copyright year: 2023
Copyright holder: The Author(s)
License: This is an Open Access article distributed under the terms of the Creative Commons Attribution License (http://creativecommons.org/licenses/by/4.0/), which permits unrestricted use, distribution, and reproduction in any medium, provided the original work is properly cited.
License URL: https://creativecommons.org/licenses/by/4.0/

Figure count: 0
Table count: 0
Page count: 11

==============================


 

Keywords: Community care, research methods, integrated care, geriatics, telemedicine, disadvantaged groups

Introduction to the theme of the conference: Research on integrated community care: Focus on GP, patients and population

Subjects: Abstract, Keynote Lectures

International keynote lecture: Models, theories, and frameworks for complex interventions: From concepts to application in primary care

Rogers Heather L.
Biocruces Bizkaia Health Research Institute , Barakaldo , Spain
CONTACT rogersheatherl@gmail.com

Subjects: Abstract, Keynote Lectures

National keynote lecture: General practice, primary healthcare and the community: linking the dots

Bastiaens Hilde
Universiteit Antwerpen , Antwerpen , Belgium
CONTACT hilde.bastiaens@uantwerpen.be

Subjects: Abstract, Poster Prize Winner

COVID-19 pneumonia in the outpatient setting in Italy: A population-based observational study

Serafini Alice
Ugolini Giulia
Palandri Lucia
Kurotschka Peter Konstantin
Scarpa Marina
Padula Maria Stella
Fornaciari Davide
Lavenia Martina
Morandi Matteo
Bellelli Francesco
Sabattini Maria Rita
Giansante Chiara
Righi Elena
Riccomi Silvia
Azienda Unità Sanitaria Locale di Modena, Local Health Authority of Modena , Modena , Italy
CONTACT alice.serafini@hotmail.it
Keywords: COVID-19, pneumonia, Italy, primarycare, observational study, electronic health record

Subjects: Abstract, SELECTED ABSTRACTS Themed topics

Pulled in two directions: The tensions between implementation and established methods to evaluate in the same project, with marginalised communities

Van Marwijk Harm
Grice-Jackson Tom
Ford Elizabeth
Musinguzi Geofrey
Gibson Linda
Van Marwijk Anne
Bastiaens Hilde
Brighton and Sussex Medical School , Brighton , United Kingdom
CONTACT H.VanMarwijk@bsms.ac.uk
Keywords: Implementation science, community health workers, mixed methods, lifestyle advice, cardiovascular risk, marginalisation

Subjects: Abstract, Selected Abstracts

A search for relevant contextual factors in intervention studies, a stepwise approach with online information.

Van Der Elst Michaël
Schoenmakers Birgitte
Schols Jos
Kemplen Gertrudis
Lepeleire Jan De
D-Scope Consortium
General Practice, ACHG – KU Leuven , Leuven , Belgium
CONTACT michael.vanderelst@kuleuven.be
Keywords: Context, online information, complex intervention, frailty, method

Subjects: Abstract, Selected Abstracts

Advance care planning among older people of Turkish origin in Belgium: an exploratory interview study

Demirkapu Hakki
Van Den Block Lieve
Maesschalck Stéphanie De
De Vleminck Aline
Colak F. Zehra
Devroey Dirk
Vrije Universiteit Brussel , Jette , Belgium
CONTACT hakki.demirkapu@vub.be
Keywords: Advance care planning, older adult, ethnicity, minority group, qualitative study

Subjects: Abstract, Selected Abstracts

Family conferences facilitating shared prioritisation and deprescibing in frail elderlies with polypharmacy cared for at home. Results from a pragmatic cluster randomised trial in primary care

Mortsiefer Achim
Wilm Stefan
Löscher Susanne
Wollny Anja
Ritzke Manuela
Drewelow Eva
Thürmann Petra
Bencheva Veronika
Abraham Jens
Meyer Gabriele
Wiese Birgitt
Icks Andrea
Montalbo Joseph
Altiner Attila
Institute of General Practice, Chair of General Practice II and Patient-Centredness in Primary Care, Department of Medicine, Faculty of Health, University Witten/Herdecke , Witten , Germany
CONTACT achim.mortsiefer@uni-wh.de
Keywords: Polypharmacy, shared decisionmaking, frailty, family conferences, cluster randomised trail

Subjects: Abstract, Selected Abstracts

A geriatric assessment intervention in primary care provided by a nurse or a general practitioner (CEPIA): a cluster-randomised trial

Orcel Veronique
Fabre Julie
Bastuji-Garin Sylvie
Renard Vincent
Boutin Emmanuelle
Caillet Philippe
Paillaud Elena
Banh Leon
Attali Claude
Audureau Etienne
Ferrat Emilie
Paris-Est Creteil University , Saint Maur Des Fosses , France
CONTACT veronique.orcel@laposte.net

Subjects: Abstract, Selected Abstracts

How does ‘home health services’ training during family medicine residency influence the medical practice of the physicians?

Unalan Pemra C.
Ercan Ezgi Yigit
Kaya Memnune Çigdem Apaydin
Temel Kübra
Department of Family Medicine, School of Medicine, Marmara University , Istanbul , Turkey
CONTACT pcunalan@gmail.com
Keywords: Home Health Services, home care, education, experience, family medicine, practice

Subjects: Abstract, Selected Abstracts

Understanding Trustful Relationships between Community Health Workers and Vulnerable Citizens during the COVID-19 Pandemic: A Realist Evaluation

Bossche Dorien Vanden
Willems Sara
Decat Peter
Ghent University , Ghent , Belgium
CONTACT dorien.vandenbossche@ugent.be
Keywords: Community health workers, primary healthcare, vulnerable populations, trust, COVID-19, realist evaluation, grounded theory

Subjects: Abstract, Selected Abstracts

Health Kiosk: Development and implementation of a low threshold community health literacy hub

Masquillier Caroline
Bastiaens Hilde
Aerts Naomi
Van Royen Kathleen
Faculty of Medicine, Primary and Interdisciplinary Care , Wilrijk , Belgium
CONTACT caroline.masquiller@uantwerpen.be
Keywords: Health Kiosk, health literacy, socioeconomically vulnerable groups, outreach working

Subjects: Abstract, Selected Abstracts

What are the determinants of older people adopting communicative e-health services? A meta-ethnography

Aslan Ayse
Armes Jo
Mold Freda
Van Marwijk Harm
University of Surrey , Guildford , United Kingdom
CONTACT a.aslan@surrey.ac.uk
Keywords: Older adults, e-health, digital technology

Subjects: Abstract, Selected Abstracts

Is self-triage by patients using a symptom checker safe?

Meer Andreas
Demurtas Jacopo
in4medicine Bern , Bern , Switzerland
CONTACT j.demurtas@in4medicine.ch
Keywords: Triage, safety, telemedicine, symptom checkers

Subjects: Abstract, Selected Abstracts

The impact of remote and telemedicine visits on family physicians’ workload

Vinker Shlomo
Cohen Avivit Golan
Green Ilan
Merzon Eugene
Yehuda Ariel
Leumit Health Services, Family Medicine , Ashdod , Israel
CONTACT vinker01@gmail.com
Keywords: Telemedicine, workload, correspondence, face-to-face

Subjects: Abstract, Clinical Topics

Are immigrants living in France more reluctant to receive vaccines than native-born French citizens? Findings from the national health Barometer study

Moussaoui Sohela
Vignier Nicolas
Sorbonne University , Paris , France
CONTACT moussaoui.sohela@gmail.com
Keywords: Hesitancy, vaccine, immunization, migrants

Subjects: Abstract, Clinical Topics

Breastfeeding mothers’ experiences with community physicians in Israel: A qualitative study

Blitman Elia
Biderman Aya
Yehoshua Ilan
Adler Limor
Israel Association of Family Physicians , Sde Yoav , Israel
CONTACT adler_l@mac.org.il
Keywords: Breastfeeding, primary care, qualitative research, ante-natal care, postpartum care

Subjects: Abstract, Clinical Topics

Clinical prediction rule for acute appendicitis in children in primary care

Blok Guus
Burger Huib
Van Der Lei Johan
Berger Marjolein
Holtman Gea
University Medical Center Groningen , Harlingen , Netherlands
CONTACT cghblok@outlook.com
Keywords: Clinical decision rule, appendicitis, children

Subjects: Abstract, Clinical Topics

Antibiotic use in ambulatory care for acutely ill children in high-income countries: A systematic review and meta-analysis

Burvenich Ruben
Dillen Hannelore
Trinh Nhung
Freer Joseph
Wynants Laure
Heytens Stefan
De Sutter An
Verbakel Jan Y.
Keywords: Anti-bacterial agents, antimicrobial resistance, drug prescriptions, primary health care, outpatients, ambulatory care, general practice, general practitioners, paediatrics, emergency medicine

Subjects: Abstract, Clinical Topics

Mild severity COVID-19 mental health implications for patients in Greece: A qualitative study

Symintiridou Despoina
Pagkozidis Ilias
Mystakidou Stavroula
Birtsou Charis
Ploukou Stella
Begou Stavroula
Andreou Martha
Dandoulakis Michael
Theodoropoulos Elias
Manolaki Chrysanthi
Avakian Ioanna
Makridou Efthymia
Avgerinou Christina
Papageorgiou Dimitra Iosifina
Smyrnakis Emmanouil
Aristotle University Primary Health Care Research Network (Auth.phc.rn.) , Thessaloniki , Greece
CONTACT authphcrn@gmail.com
Keywords: COVID-19, mental health, primary health care, Greece

Subjects: Abstract, Clinical Topics

Sociodemographic characteristics and cardiovascular events in patients with severe mental disorders

Tabueña Noemí Olona
Martín María Isabel Fernández San
Calafell Javier Molero
Gatius Jordi Real
Sanchez Miguel Castillo
Tejon Susana González
Lopez Luis Miguel Martin
Idiap Jordi Gol, Unitat Suport Recerca Barcelona ciutat , Barcelona , Spain
CONTACT mifsanmartin.bcn.ics@gencat.cat
Keywords: Severe mental disorders, cardiovascular incidence, sociodemografic factors

Subjects: Abstract, Clinical Topics

Real life HbA1c variability profiles and association with ASCVD risk in T2DM

Lapidus Alon
Adler Limor
Maccabi Healthcare Services , Lehavim , Israel
CONTACT alon.lap@gmail.com
Keywords: Diabetes millitus, HbA1c, variability profiles, ASCVD

Subjects: Abstract, Clinical Topics

Implementation of a lifestyle programme in primary care among cancer survivors: Lessons learned so far

Huizinga Famke
Westerink Nico-Derk Lodewijk
Berendsen Annette J.
Walenkamp Annemiek M. E.
De Greef Mathieu H. G.
De Bock Geertruida H.
Berger Marjolein Y.
Brandenbarg Daan
University Medical Center Groningen , Groningen , Netherlands
CONTACT f.huizinga@umcg.nl
Keywords: Lifestyle, physical activity, cancer survivors, primary care, implementation

Subjects: Abstract, Clinical Topics

Identifying and prioritising do-not-do recommendations in Dutch general practice

Burgers Jako
Van Dulmen Simone
Kool Tijn
Dutch College of General Practitioners , Utrecht , Netherlands
CONTACT j.burgers@nhg.org
Keywords: Family practice, Netherlands, clinical practice guidelines, low-value care, de-implementation

